# Direct bony invasion of malignant melanoma

**DOI:** 10.4103/0019-5413.55469

**Published:** 2009

**Authors:** Viswanath Mula, Adhip Mandal, Edward Britton, Vaidyanathan Shiv Shanker

**Affiliations:** Colchester General Hospital, Colchester, CO4 5JL, United Kingdom

**Keywords:** Bone tumor, metastatic melanoma, malignant melanoma, direct invasion

## Abstract

Malignant melanoma is known to spread by local extention, by the lymphatics by the blood stream. Direct invasion of the bone from a cutaneous melanoma is unknown. Hence, this case is presented in view of its rarity. A 75-year-old Caucasian lady presented with a small papillary lesion in the region of a recurrent chronic cellulitis on the lower third of the lateral aspect of the right leg. Histopathology diagnosed the lesion as locally advanced malignant melanoma. Radiological investigations by X-ray and magnetic resonance imaging revealed malignant infiltration of the tibia in its mid and lower third with two soft tissue metastatic masses adjacent. Histology following amputation confirmed malignant melanoma with cranial resection margin involvement. She underwent a further above-knee amputation followed by chemotherapy. The patient recovered from the amputation but subsequently died 6 months later due to bronchopneumonia from lung metastasis.

## INTRODUCTION

Cutaneous melanoma is a malignant neoplasm arising from epidermal melanocytes. The earliest description is in the writings of Hippocrates in fifth century BC.^1^ Metastasis from malignant melanoma is known to spread by local extension, by the lymphatics, or by the bloodstream. Blood-borne distant metastases of melanoma are seen in the lungs, gastrointestinal tract, brain, parotid, heart, and skin, but rarely in the bones. The main cause of mortality in malignant melanoma is from the secondaries that may occur many years after excision of the primary lesion. Local extension is centrifugal via dermal lymphatic permeation into the surrounding skin. Bone secondaries are usually from breast, bronchus, thyroid, kidney, and prostrate cancers.

A 75-year-old Caucasian lady presented with recurrence of chronic cellulitis on the lower third of the lateral aspect of the right leg. The cellulitis had been on going for the past 10 years. The current symptoms started 2 months before presentation when she noticed a small papillary lesion, which was biopsied. The histopathology result stated that the lesion was a locally advanced malignant melanoma. During her admission, the lesion progressively increased in size over 2 weeks.

**Figure 1:**
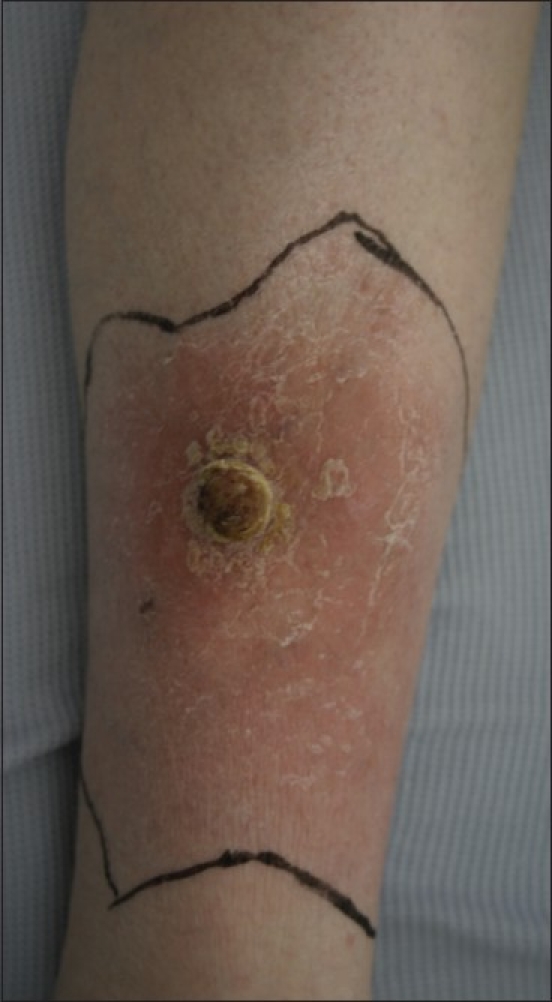
Photograph of right lower leg with papillary lesion and adjacent cellulitis

On examination, she was thin but not cachectic. She had a 2 × 2 cm yellow papillary lesion with a surrounding 4 cm red indurated area over the lower third of the right leg on the lateral aspect [Figure 1]. There were no satellite skin lesions and no palpable lymph nodes.

**Figure 2:**
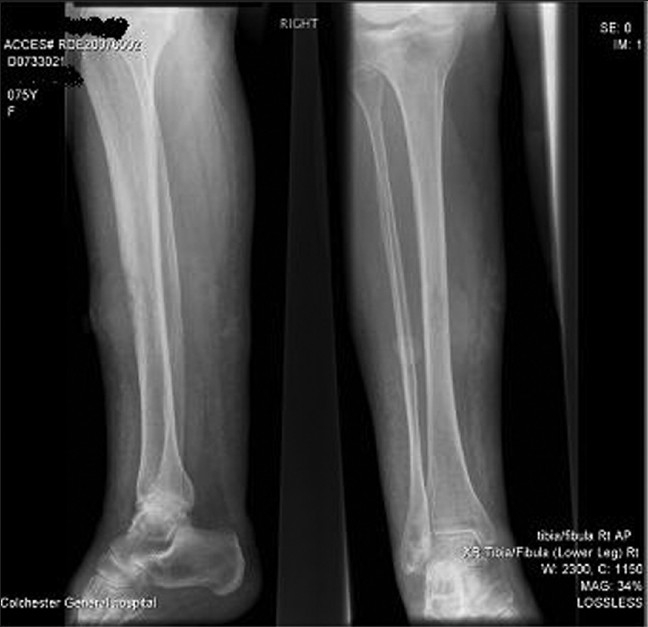
Plain radiograph of right tibia and fibula showing cortical erosion of lower third of tibia

**Figure 3:**
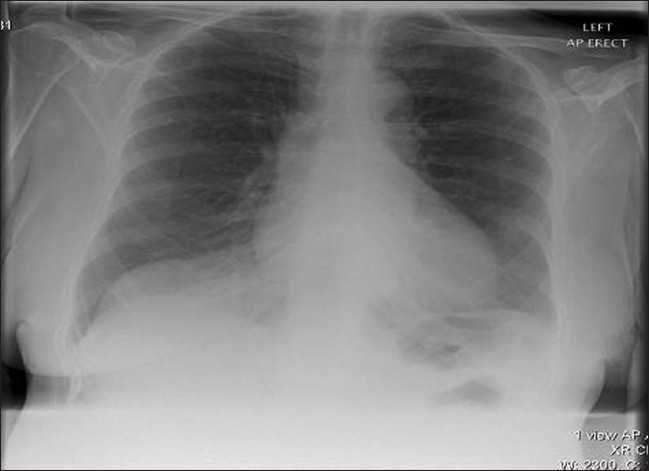
Chest X-ray: Normal with no signs of metastatic lesions

**Figure 4:**
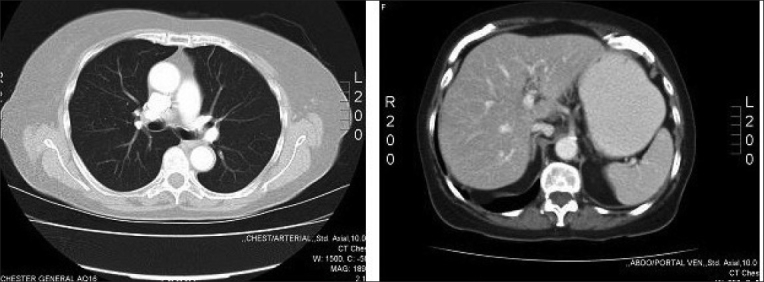
CT scan of chest and abdomen: Normal with no signs of metastatic lesions

**Figure 5:**
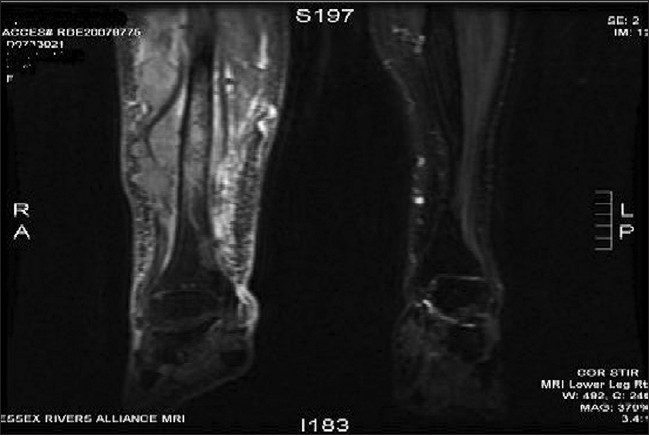
MRI of right leg showing malignant infiltration of tibia in its mid and lower third with adjacent soft tissue masses

**Figure 6:**
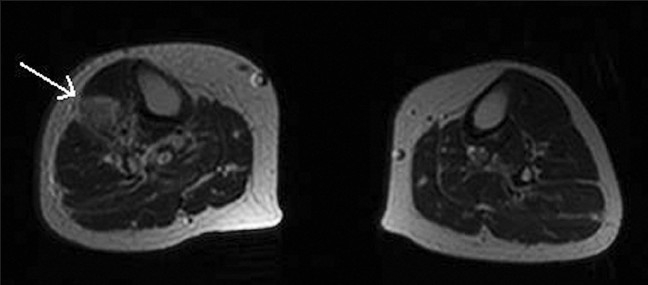
Axial MRI section of right leg showing extent of lesion infiltration down to the tibia

**Figure 7:**
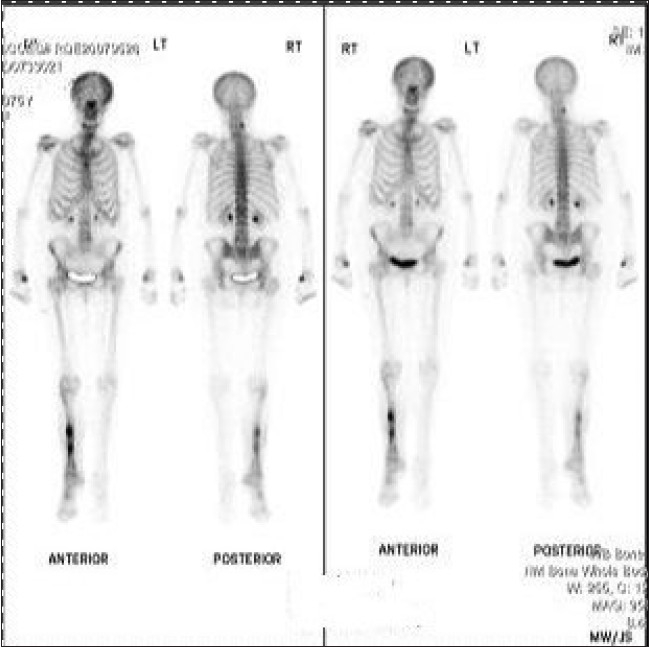
Tc99MDP scan showing hot spots in right tibia

A plain radiograph [Figure 2] revealed erosion lesions on the right tibia suggesting bone invasion. Her white cell count was 23.9 × 10^6^ (normal 4.0–11.0) and C-reactive protein was 128 mg/L (normal 0–10). Full blood count, urea, electrolytes, liver function tests, and clotting profile were within the normal range. Chest radiograph [Figure 3], ultrasound scan and computed tomography (CT) chest, abdomen, and pelvis [Figure 4] did not reveal any metastases or lymph node involvement. Magnetic resonance imaging [Figures 5 and 6] of the right leg confirmed malignant infiltration of the tibia in its mid and lower third with two further soft tissue masses cranial to this consistent with soft tissue metastases. The Tc99 MDP scan [Figure 7] revealed no other bony hot spots apart from the right tibia.

She initially underwent a below-knee amputation at the Regional Bone Tumour Unit. Histology confirmed malignant melanoma involving adjacent soft tissue with infiltration into periosteum of the bone and cranial resection margin involvement [Figure 8]. The amputation was therefore extended above the knee. She subsequently underwent adjuvant chemotherapy. Later, she developed subcutaneous nodules in the epigastrium and lung metastasis over the next 4 months. Unfortunately, she died from pneumonia and advanced metastatic cancer at a hospice 6 months later.

**Figure 8a:**
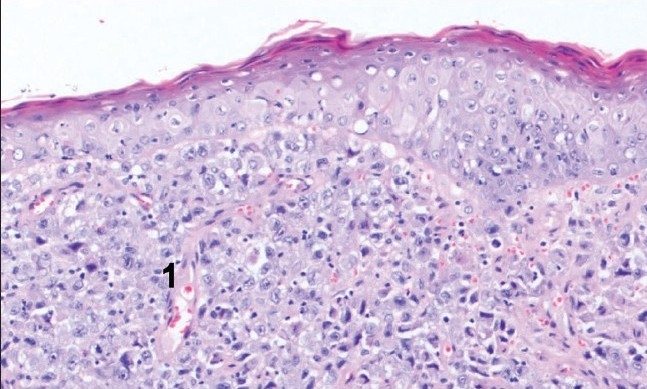
H and E section showing malignant melanocytes infiltrated into subcutaneous tissues (1)

**Figure 8b:**
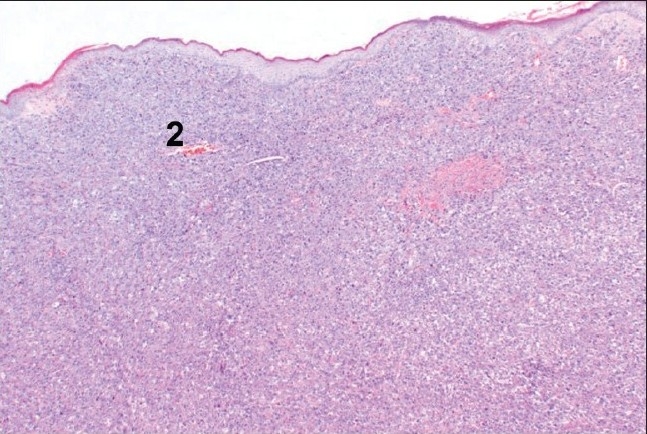
H and E section showing maignant melanocytes in periosteum of bone (2)

## DISCUSSION

Malignant melanoma has been considered a rare tumor with an unpredictable natural history. The rate of increase in the incidence of melanoma is greater than for any other cancer in Caucasians, with the exception of bronchogenic carcinoma. It is likely that between a third and a half of all melanomas develop in a benign naevus of many years' standing, which could be the case in our patient. The most common site for females is the lower leg, as in our patient. This is the first case of malignant melanoma with direct invasion of bone in the literature [Table 1].

**Table 1:** Rare tumors of the bone

Primary	Site	Author	Year
Melanoma	Tibia	Mula *et al*.	Aug 2009
Endometrial carcinoma	Tibia	Ishibashi *et al*.^10^	Mar 2007
Recurrent basal cell carcinoma	Tibia	Perez de la Fuente *et al*.^11^	Jan-Feb 2006
Colon cancer	Hand	Gamblin *et al*.^12^	Jan 2006
Epitheloid hemangioendothelioma	Phanlanx	Kitagawa *et al*.^13^	May 2005
Hyalinizing clear cell carcinoma	Hard palate	Chao *et al*.^14^	May 2004
Fibrous histiocytoma	Distal radius	Jebson *et al*.^15^	Mar 2004
Clear cell sarcoma	Capitate	Reichert *et al*.^16^	Nov 2001
Intraosseous lipoma	Calcaneus	Burd *et al*.^17^	Mar 2001
Thymic tumor	Ring finger	Shannon *et al*.^18^	Nov 2000
Plasmacytoma	Mastoid	Engelsma *et al*.^19^	May 2000
Paraganglionoma	Sacrum	Coles *et al*.^20^	Apr 2000
Neurilemmoma	Radius	Gine *et al*.^21^	Mar 2000
Pseudo anaplastic giant cell tumor	Sacrum	Layfield *et al*.^22^	Feb 1999

The development of a malignancy in a mole should be suspected if any of the following changes occur:

**Table:** 

**Major signs**	**Minor signs**
Change in size	Inflammation
Change in shape	Crusting or bleeding
Change in color	Sensory change, e.g. itch
	Diameter 5 mm or more

Suspicious lesions should be removed completely with a 2 mm margin; use of incision or punch biopsies is deprecated because accurate histological staging is impossible and the treatment is dependent on the histology. In our patient, punch biopsy was performed before diagnosing melanoma. A planned excision was performed when bony involvement was found.

A complete staging workup, including examination of the skin and mucous membranes; CT scans of the chest, abdomen, and pelvis; and a bone scan, should be performed.^2^,^3^ There is an emerging role for positron-emission tomography in the staging of malignant melanoma.^4^–^6^

Radical surgical treatment is favored by many authors, with acceptable mortality and morbidity rates in gastrointestinal metastasis.^7^,^8^ We opted for radical surgical treatment as we do not have sufficient data on bony malignant melanoma. If there is a single site of metastatic disease, surgery is sometimes employed and there is a study examining the use of an adjuvant vaccine (“cancervax”) following “metastasectomy”.^9^ There is no long-term follow-up or 5-year survival data available on bony metastatic melanoma.

In conclusion, the principles of treatment are proper staging and radical surgery where appropriate.

## References

[1] Williams N, Bulstrode C, Russell RC (2004). Bailey and Love's short practice of surgery.

[2] Prayson A, Sebek BA (2000). Parotid gland malignant melanomas. Arch Pathol Lab Med.

[3] Ozyuncu N, Sahin M, Altin T, Karaoguz R, Guldal M, Akyurek O (2006). Cardiac metastasis of malignant melanoma: A rare cause of complete atrioventricular block. Europace.

[4] Gulec SA, Fanes MB, Lee CC, Kirgan D, Glass C, Morton DL (2003). The role of fluorine-18 deoxyglucose positron emission tomography in the management of patients with metastatic melanoma: Impact on surgical decision making. Clin Nucl Med.

[5] Ludwig V, Komori T, Kolb D, Martin WH, Sandler MP, Delbeke D (2002). Cerebral lesions incidentally detected on 2-deoxy-2-(18F]fluoro-D-glucose positron emission tomography images of patients evaluated for body malignancies. Mol Imaging Biol.

[6] Jacob A, Brightman RP, Welling DB (2007). Bilateral cerebellopontine angle metastatic melanoma: A case report. Ear Nose Throat J.

[7] Marin M, Vlad L, Grigorescu M, Sparchez Z, Dumitra D, Muti L (2002). Metastasis of malignant melanoma in the small intestine: A case report. Rom J Gastroenterol.

[8] Agrawal S, Yao TJ, Coit DG (1999). Surgery for melanoma metastatic to the gastrointestinal tract. Ann Surg Oncol.

[9] Russell LA (2003). Melanoma treatment. Patient Care.

[10] Ishibashi M, Fujiwaki R, Nakayama I, Miura H, Sawada K (2007). Endometrial carcinosarcoma presenting as a tibial metastases. Int J Clin Oncol.

[11] Perez de la Fuente T, Gonzalez Gonzales I (2006). Recurrent basal cell carcinoma of the lower limb with tibial invasion. J Cutan Med Surg.

[12] Gamblin TC, Santos RS, Baratz M, Landreneau RJ (2006). Metastatic colon cancer to the hand. Am Surg.

[13] Kitagawa Y, Ito H, Iketani M, Hirukawa M, Yokoyama M, Maeda S (2005). Epithelioid hamangioendothelioma of the phalanx. J Hand Surg Am.

[14] Chao TK, Tsai CC, Yeh SY (2004). Hyalinizing clear cell carcinoma of the hard palate. J Laryngol Otol.

[15] Jebson PJ, Sullivan M, Murray PM (2004). Malignant fibrous histiocytoma of the distal radius. J Hand Surg Am.

[16] Reichert B, Hoch J, Plotz W, Mailänder P, Moubayed P (2001). Metastatic clear-cell sarcoma of the capitates. J Bone Joint Surg Am.

[17] Burd TA, Reddy R, Greene WB (2001). Intraosseus lipoma of the calcaneus. Orthopedics.

[18] Shannon FJ, Antonescu CR, Athanasian EA (2000). Metastatic thymic carcinoma in a digit: A case report. J Hand Surg Am.

[19] Engelsma RJ, De Bree R, Janssen JJ (2000). Plasmacytoma of the mastoid bone: solitary and systemic. J Laryngol Otol.

[20] Coles CP, Alexander DI, Gross M, Holness RO, Covert AA, Murray SK (2000). Intraosseus paraganglioma of the sacrum: A case report. Can J Surg.

[21] Gine J, Calmet J, Sirvent JJ, Doménech S (2000). Intraosseus neurilemmoma of the radius: A case report. J Hand Surg Am.

[22] Layfield LJ, Bentley RC, Mirra JM (1999). Pseudoanaplastic giant cell tumor of bone. Arch Pathol Lab Med.

